# Commentary: α-Synuclein Interacts with Lipoproteins in Plasma

**DOI:** 10.3389/fnmol.2017.00362

**Published:** 2017-11-02

**Authors:** Andrei Surguchov

**Affiliations:** Department of Neurology, Kansas University of Medical Center Research Institute, Kansas City, MI, United States

**Keywords:** alpha-Synuclein, high density lipoproteins, apolipoprroteins, ApoA-1, APOE

Recent publications suggest that α-synuclein for a long time associated with neuronal functions and neurodegenerative diseases in fact might be positioned on a crossroad between synaptic functions and lipid metabolism. Hydrophobic core of α-synuclein including a cholesterol binding domain at residues 67–78 (Fantini et al., 2011) may be responsible for lipid interaction, whereas more hydrophilic regions are involved in the synaptic role. Previous studies with systematic mutagenesis of α-synuclein revealed distinct sequence requirements for physiological synaptic functions and other activities of α-synuclein (Burré et al., 2012).

α-Synuclein is a small (~14 KDa) predominantly neuronal protein implicated in Parkinson's disease and other neurodegenerative disorder, i.e., dementia with Lewy bodies, and multiple system atrophy collectively called synucleinopathies (Goedert et al., 2017). α-Synuclein and two other members of the family, β- and γ-synuclein are expressed only in eukaryotes and share structural resemblance to apolipoproteins (Clayton and George, 1998; Auluck et al., 2010). Synucleins are structurally unfolded proteins possessing considerable structural plasticity and located both in cytoplasm and cellular organelles (Uversky et al., 2001; Surgucheva et al., 2006; Surguchov, 2015). α-Synuclein is prone to aggregate and form intracellular inclusions of various size, including relatively small toxic oligomeric aggregates and large megadalton α-synuclein-rich deposits—Lewy bodies, the hallmarks of Parkinson's disease (Spillantini et al., 1997). Investigation of α-synuclein comes across with several unexpected findings, showing that its properties are different from that of the majority of typical intracellular globular proteins. Although α-synuclein does not contain N-terminal signal peptide, it can be translocated from cells into the extracellular space and is present in human body fluids, including the cerebrospinal fluid and blood plasma (El-Agnaf et al., 2006). Furthermore, aggregated forms of α-synuclein spread throughout neighboring portions of the brain by propagation in a prion-like mode (Olanow and Brundin, 2013). Another feature of α-synuclein which require further studies is that it penetrates through the blood-brain barrier (BBB) by an unclear mechanism (Sui et al., 2014; Peelaerts et al., 2015).

The results of recent studies demonstrate that unusual behavior of α-synuclein, at least in some cases, may be explained by its interaction with apolipoproteins. Apolipoproteins are amphipathic proteins binding lipids to form lipoproteins and transporting them through the lymphatic and circulatory systems. Many studies in genetics, cell biology, and biochemistry provided substantive insights into the causal relationship between lipids and atherogenesis (Brown and Goldstein, 1986; Willer et al., 2013).

Although the role of lipoproteins in the penetration of α-synuclein through BBB is not systematically investigated, previous studies have demonstrated that low-density lipoprotein receptor-related protein-1 (LRP-1) may be involved in α-synuclein crossing through the blood-brain barrier (Sui et al., 2014).

Interestingly, this interaction with plasma apolipoproteins is highly specific. For example, α-synuclein easily binds with apoE, apoJ, and apoA1, but not with apoB and is present in high density lipoprotein (HDL) fraction in plasma. A complex between α-synuclein and ApoJ is also found in the intermediate fraction between HDL and low density lipoproteins (LDLs), referred to as lipoprotein (a) or Lp(a) (Emamzadeh and Allsop, 2017). ApoE modulates α-synuclein aggregation depending on apoE concentration; at concentration lower than 15 nM all apoE isoforms enhance aggregation, whereas at higher concentration the aggregation is reduced (Emamzadeh et al., 2016).

Furthermore, α-synuclein itself is able to form lipid-protein nanometer-sized particles reminiscent of HDL with a diameter of 23 nm composed of α-synuclein and anionic phospholipids (Eichmann et al., 2016, 2017). Importantly, at high protein-to-lipid ratios, α-synuclein restructures large lipid vesicles into lipoprotein nanoparticles with diameter 7–10 nm, resembling those formed by lipoproteins (Varkey et al., 2013). Investigation of the structure of α-synuclein-lipid nanoparticles by circular dichroism, cryo-electron microscopy, fluorescence spectroscopy, and other methods have demonstrated that they have an overall ellipsoidal architecture similar to that reported for apolipoproteins. In these complexes, α-synuclein forms helical oligomers and assumes a shorter broken helical structure, indicating that in nanoparticles its interaction with lipid is different from its interaction with liposomes. These data suggest that α-synuclein has the ability to function as an apolipoprotein-like, intracellular lipid-carrying protein (Varkey et al., 2013). Binding of α-synuclein with apolipoproteins not only changes its properties, but also affects cholesterol metabolism (Barceló-Coblijn et al., 2007). These new results may point to the existence of a novel link between neurodegenerative diseases, in which α-synuclein is implicated, and defects in lipid metabolism and apolipoproteins associated with atherosclerosis and hyperlipidemia. α -Synuclein-related nanoparticles may play an important role in lipid and fatty acid transport functions in healthy conditions and in diseases. Both synucleins and apolipoproteins are present in plasma as well as in erythrocytes, platelets, lymphocytes, exosomes, and it is a matter of further investigation to identify where their interaction takes place. Further investigation will unwrap the intimate details of synuclein-apolipoprotein communication.

## Author contributions

The author confirms being the sole contributor of this work and approved it for publication.

### Conflict of interest statement

The author declares that the research was conducted in the absence of any commercial or financial relationships that could be construed as a potential conflict of interest.
